# A Sensor Array Realized by a Single Flexible TiO_2_/POMs Film to Contactless Detection of Triacetone Triperoxide

**DOI:** 10.3390/s19040915

**Published:** 2019-02-21

**Authors:** Xiaorong Lü, Puqi Hao, Guanshun Xie, Junyuan Duan, Li Gao, Bingxin Liu

**Affiliations:** 1 Qinghai Provincial Key Laboratory of New Light Alloys, Qinghai Provincial Engineering Research Center of High Performance Light Metal Alloys and Forming, Qinghai University, Xining 810016, China; jwplxr@sina.com (X.L.); pheghost@outlook.com (P.H.); guanshunxie@126.com (G.X.); 2 State Key Laboratory of Material Processing and Die & Mould Technology, School of Material Sciences and Engineering, Huazhong University of Science and Technology (HUST), Wuhan 430074, China

**Keywords:** TATP, TiO_2_, sensor array, POMs, gas sensitivity

## Abstract

The homemade explosive, triacetone triperoxide (TATP), is easy to synthesize, sensitive to detonation but hard to detect directly. Vapor sensor arrays composed of a few sensor materials have the potential to discriminate TATP, but the stability of the sensor array is always a tricky problem since each sensor may encounter a device fault. Thus, a sensor array based on a single optoelectronic TiO_2_/PW_11_ sensor was first constructed by regulating the excitation wavelength to discriminate TATP from other explosives. By in situ doping of Na_3_PW_12_O_40_, a Keggin structure of PW_11_ formed on the TiO_2_ to promote the photoinduced electron-hole separation, thus obviously improving the detection sensitivity of the sensor film and shortening the response time. The response of the TiO_2_/PW_11_ sensor film to TATP under 365, 450 and 550 nm illumination is 81%, 42%, and 37%, respectively. The TiO_2_/PW_11_ sensor features selectivity to TATP and is able to detect less than 50 ppb. The flexibility and stability of the flexible sensor film is also demonstrated with the extent of bending. Furthermore, the sensing response cannot be affected by ambient air below 60% relative humidity.

## 1. Introduction

In recent years, explosive-based terrorism has grown extensively because explosive weapons have a simple manufacturing process, are easy to dispose of, and can cause enormous injury [1]. Peroxide-based explosives, such as triacetone triperoxide (TATP) [2], are the most powerful and dangerous and the first choice for terrorism all around the world. TATP has very high vapor pressure and is very sensitive to heat, impact, and explosion shock. Therefore, the development of sensor devices for explosive detection has great potential. As shown in Table S1, a few technologies, such as ion mobility spectrometry (IMS) [3,4], electrochemical method [5,6], colorimetric sensor [7,8] and gas sensing sensors [9,10,11,12], have been discovered in the field of trace TATP detection. IMS converts trace vapor into ions at atmospheric pressure and characterizes the gas phase mobility of these ions in a weak electric field to offer a highly efficient response to trace gas or vapor species. However, IMS equipment is expensive. The electrochemical method is always employed in a solution system. The colorimetric sensor possesses good accuracy but is very time consuming. Gas sensing sensors involve two key functions: (i) identification of a target gas via a gas–solid interaction, which causes an electron change of the oxide surface and (ii) conversion of the surface phenomena into resistance or current change of the sensor, called transducer function. Among them, the gas sensor is one of the most widely used means in the market because of its advantages of quick response, high sensitivity, good stability, simple use, low cost, and so on.

In the field of sensors, TiO_2_-semiconductor nanocrystals [13,14,15,16,17,18] become a flash point in the research of sensors owing to their large unique surface area, excellent optical response, excellent chemical stability, good biocompatibility and one-dimensional electron transport structure. However, the relatively wide band gap (3.2 eV) and low photoinduced charge carrier separation efficiency [19] hinder the practical application of TiO_2_. To solve this problem, here we are proposing to develop a sensor based on polyoxometalates (POMs)-loaded TiO_2_-semiconductor nanocrystal arrays.

POMs are composed of cations and polyanion clusters with structural diversity [20]. POMs are usually deemed to electron reservoirs because they have a strong ability to bear electrons and deliver electrons, illustrating their available redox nature [21]. So far, many classic paradigms of POMs, such as Keggin and Dawson structures, have been reported [22,23,24,25].

However, a single electrical sensor has difficulty in meeting the actual needs of qualitative identification of different explosives. In addition, the sensitivity and selectivity of single sensor are still a bottleneck for current solid sensors of gases. Naaman [26] developed an array of sensors based on non-specific interactions, which is capable of detecting TATP with high selectivity. Therefore, a sensor array is needed to make explosive identification possible. Nevertheless, the stability of the sensor array is always a tricky problem since each sensor may encounter a device fault. It would be very attractive if the function of a sensor array can be realized by only employing one sensor.

In this work, the regulating of the sensing properties of TiO_2_ is achieved by the doping of POMs (sodium phosphotungstate, Na_3_O_40_PW_12_), which dominates the surface states effectively in the Keggin structure to form TiO_2_/PW_11_O_39_ (denoted as TiO_2_/PW_11_ hereafter) and form a sensor array through the illumination of different wavelengths. We characterized the structure and morphology of the TiO_2_/PW_11_ nanocrystals in detail and tested their properties, gaining insight into their sensitivity to the TATP explosive. In addition, we compared with pure TiO_2_ and revealed the reason of the performance enhancement of the sensor doped with POMs.

## 2. Materials and Methods

### 2.1. Materials

Sodiumphosphotungstate (Na_3_O_40_PW_12_), isopropyl alcohol (IPA), titanium tetraisopropoxide (TTIP) were of analytical grade. Standard distilled water was used in all experiments, sodium was an authenticated material purchased from the State Bureau of Technical Supervision, China.

### 2.2. Synthesis of TiO_2_ and TiO_2_/PW_11_ Nanocrystals

The pure TiO_2_ was synthesized by 100 μL of titanium tetraisopropoxide (TTIP) diluted with 900 μL of isopropanol, and 708 μL of this solution was dripped with vigorous stirring into 29.3 mL distilled water at room temperature. After 20 h at 170 °C in a 40 mL Teflon-lined 316 stainless-steel reaction vessel, the solution was completely transparent and colorless. Then, the 30 mL solution was centrifuged using distilled water and dried at 60 °C to finally obtain pure TiO_2_ powder.

The TiO_2_/PW_11_ was synthesized as described by Weinstock [27]. A total of 100 μL of TTIP was diluted with 900 μL of isopropanol, and 708 μL of this solution dripped with vigorous stirring into 367 mg (0.12 mmol) of Na_3_O_40_PW_12_ in 29.3 mL distilled water at room temperature. This gave 30 mL of a turbid (cream white) mixture, with a final concentration of 8 mM Ti (IV) and 4 mM Na_3_O_40_PW_12_. After 20 h at 170 °C in a 40-mL Teflon-lined 316 stainless-steel reaction vessel, the solution was completely transparent and colorless with no precipitate. For isolation and purification of TiO_2_/PW_11_ nanocrystals, the 30 mL solution was centrifuged using distilled water and NaCl because the primary POMs are fully soluble in NaCl solution. Four additional cycles of precipitation by adding NaCl, followed by centrifugation and redissolution in distilled water, eventually determined that trace amounts of POMs by-product were no longer present.

### 2.3. Characterization

Transmission electron microscopy (TEM) and Energy dispersive X-ray (EDS) mapping images was carried out on a JEM-2100F microscope. X-ray diffraction (XRD) patterns were measured using a Bruker D8 Advance X-ray diffractometer at a scanning rate of 6° min^−1^ with 2θ ranging from 20° to 80°, using CuKα radiation (λ = 1.5418 Å). ^31^P NMR spectra were recorded on a DRX-500 MHz (Bruker) spectrometer. FTIR spectra were recorded on a Magna 560 FT-IR spectrometer. UV/Vis absorption spectra were recorded on a Shimadzu UV-2550 UV/Vis spectrometer in the range 200–800 nm. An X-ray photoelectron spectroscopy (XPS, PHI5000 ESCA, Perkin Elmer, Waltham, MA, USA) equipped with an Al Kα source (1486.6 eV photons) was used to characterize the doping of POMs in TiO_2_.

### 2.4. Device Fabrication and Gas Sensing Properties Testing

Interdigital gold electrodes are the hyperfine circuits obtained by electrochemical processing on polyimide (PI) substrate. The sensor film was constructed by dispersing TiO_2_/PW_11_ nanocrystals with different molar feed ratios into THF. Uniformly dripping onto the surface of the interdigital electrode, naturally drying, and repeating the above steps to obtain the TiO_2_/PW_11_ film. The thickness of the film was controlled by the dripping cycles. The sample was dried naturally in air overnight. 

The different analytes were evaporated in a 50 mL transparent chamber and the test was conducted at room temperature in saturated 2,4,6-trinitrotoluene (TNT), 2,4-dinitrotoluene (DNT), picric acid (PA), hexogen (RDX), and 2,4,6-trinitrophenylmethylnitramine (Tetryl) vapor. Since TATP has very high vapor pressure at room temperature, it was diluted using air to 600 ppb. The time-dependent photoresponse of the sensor film was conducted in a conventional two electrode configuration and recorded by a Keithley 4200A-SCS Parameter Analyzer under 365, 450 and 550 nm monochromatic light. Thus, the sensor array based on the single optoelectronic TiO_2_/PW_11_ sensor was constructed by regulating the excitation wavelength (365, 450 and 550 nm).

## 3. Results and Discussion

### 3.1. Synthesis and Characterization of TiO_2_/PW_11_ Nanocrystals

**Figure 1:**
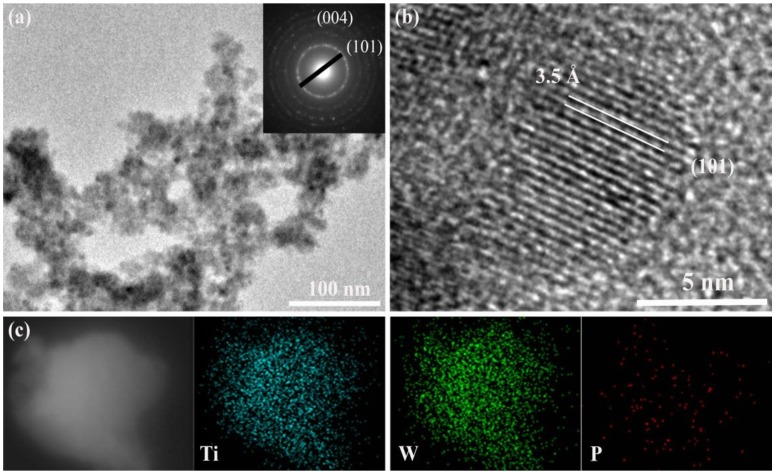
Characterization of TiO_2_/PW_11_ nanocrystals. (**a**) TEM image of TiO_2_/PW_11_ nanocrystals; inset: selected-area electron diffraction pattern of the particles. (**b**) HRTEM image of TiO_2_/ PW_11_ nanocrystals with fringes corresponding to (101) planes. (**c**) EDS elemental mapping images of Ti, W and P.

TiO_2_/PW_11_ nanocrystals were obtained by hydrothermal reaction of titanium tetraisopropoxide, isopropanol and Na_3_O_40_PW_12_ aqueous solution in distilled water for 20 h at 170 °C. TEM images of TiO_2_/PW_11_ nanocrystals (Figure 1a) show an average particle size of 5.9 ± 1.4 nm. Electron diffraction of the particles featured well-defined rings (Figure 1a inset), indicating that it is crystal structure. Of which, high-resolution TEM (HRTEM) images of the nanocrystals brings insight into the exposed (101) facets. Energy dispersive X-ray (EDS) mapping images (Figure 1c) display the presence and homogeneous distribution of titanium, tungsten and phosphorus elements in TiO_2_/PW_11_ samples. These results confirm the formation of TiO_2_/PW_11_ composite.

**Figure 2:**
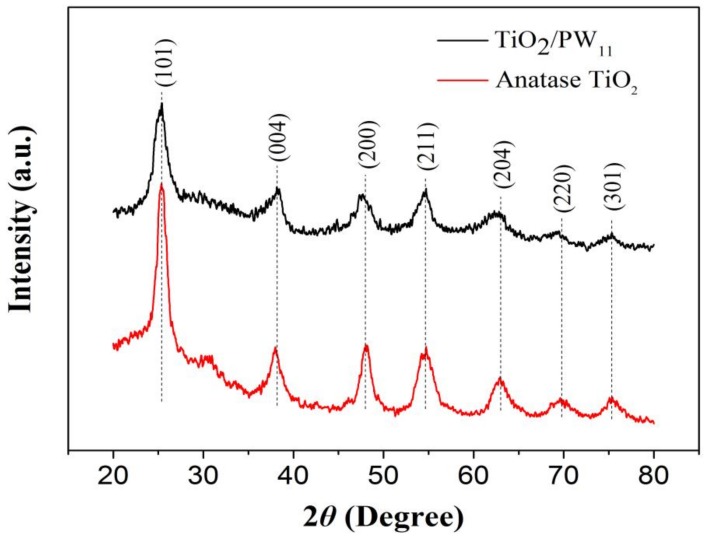
XRD patterns of the anatase TiO_2_ and TiO_2_/PW_11_.

To consider the crystalline phases and the crystallinity of the TiO_2_/PW_11_ nanocrystals, X-ray diffraction (XRD) characterization was conducted (Figure 2). It is worth mentioning that the crystalline structure of the TiO_2_/PW_11_ remained in anatase phases TiO_2_ since diffraction peaks are observed at 25.3°, 37.8°, 48.0°, 55.1°, and 62.7° [28,29]. However, the diffractions originated from the interaction between POMs and TiO_2_ are hard to observe; this might be attribute to the interaction of phosphotungstate in the octahedral interstitial site or the substitutional position of TiO_2_. These results indicated the phosphotungstate might interact with titania through oxygen atoms and that one W atom in Na_3_O_40_PW_12_ was substituted to form the Keggin structure of TiO_2_/PW_11_ [30]. Moreover, Debye–Scherrer analysis of XRD data gave an anatase–crystallite size of 4.89 ± 0.2 nm, basically identical to the average particle size obtained from TEM images.

**Figure 3:**
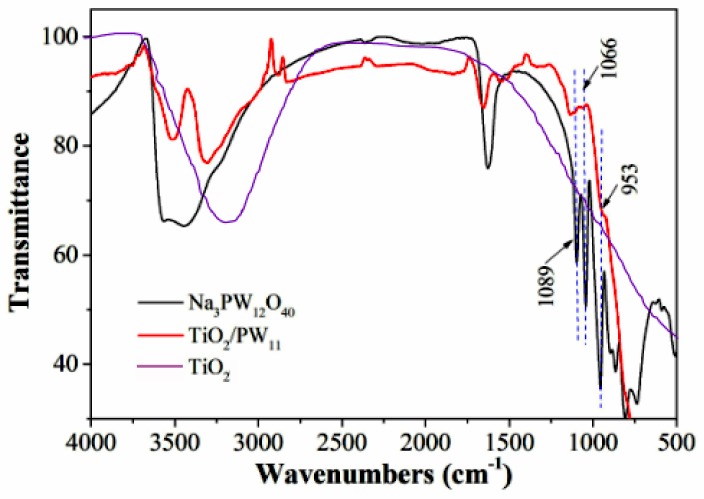
FTIR spectra of Na_3_PW_12_O_40_, TiO_2_/PW_11_ and TiO_2_.

The Keggin type structure of PW_11_ formed in TiO_2_ nanocrystals was further proved by FTIR (Figure 3). The characteristic bands observed at 1089, 1066, and 953 cm^−1^ in TiO_2_/PW_11_ nanocrystal attributes to vibrations of P-O, W-O-W and W-O [29], which are not found in TiO_2_/PW_11_ nanocrystals. These results prove the Keggin PW_11_ has been successfully modified on TiO_2_.

**Figure 4:**
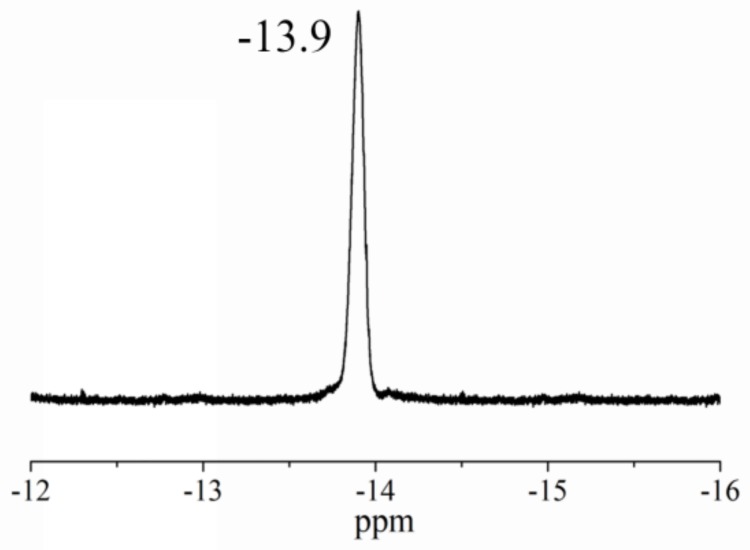
Solid-state ^31^P NMR spectra of TiO_2_/PW_11_ nanocrystals.

Solid-state ^31^P CPMAS NMR spectroscopy of TiO_2_/PW_11_ nanocrystals showed a single broad signal Keggin unit at δ= −13.9 ppm (Figure 4). Thus showing the formation of a single phosphorus-containing species and that the molecular structure in the solid state was retained in the sample [28].

**Figure 5:**
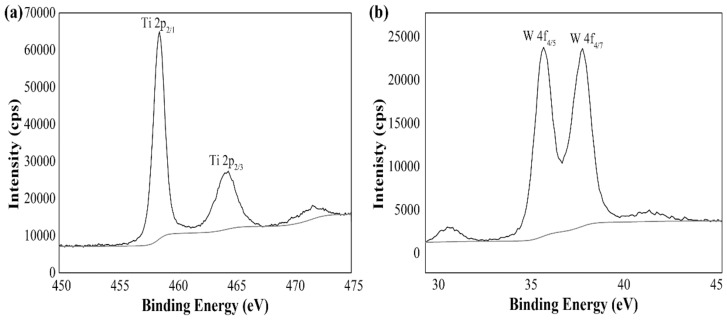
XPS spectra of Ti (**a**) and W (**b**).

The POMs deposited onto a TiO_2_ were also analyzed by X-ray photoelectron spectroscopy (XPS). As shown in Figure 5, the binding energy (BE) of Ti 2p_2/1_ and Ti 2p_3/2_ from Ti^4+^ can be detected at 458.54 eV and 464.39 eV [31]. The XPS spectra of W 4f_5/2_ and W 4f_7/2_ show the BE at 35.38 and 37.49 eV [31]. This is further proof for the adsorption of PW_11_ onto the TiO_2_. Moreover, the XPS measurements were performed to reveal the atomic content (Table S2). Based on the peak areas of W and Ti, it can be concluded that approximately 3.94% of the W is on the surface of the TiO_2_ (the content of TiO_2_ is 17.6%).

### 3.2. Optoelectronic Gas Sensor Properties

**Figure 6:**
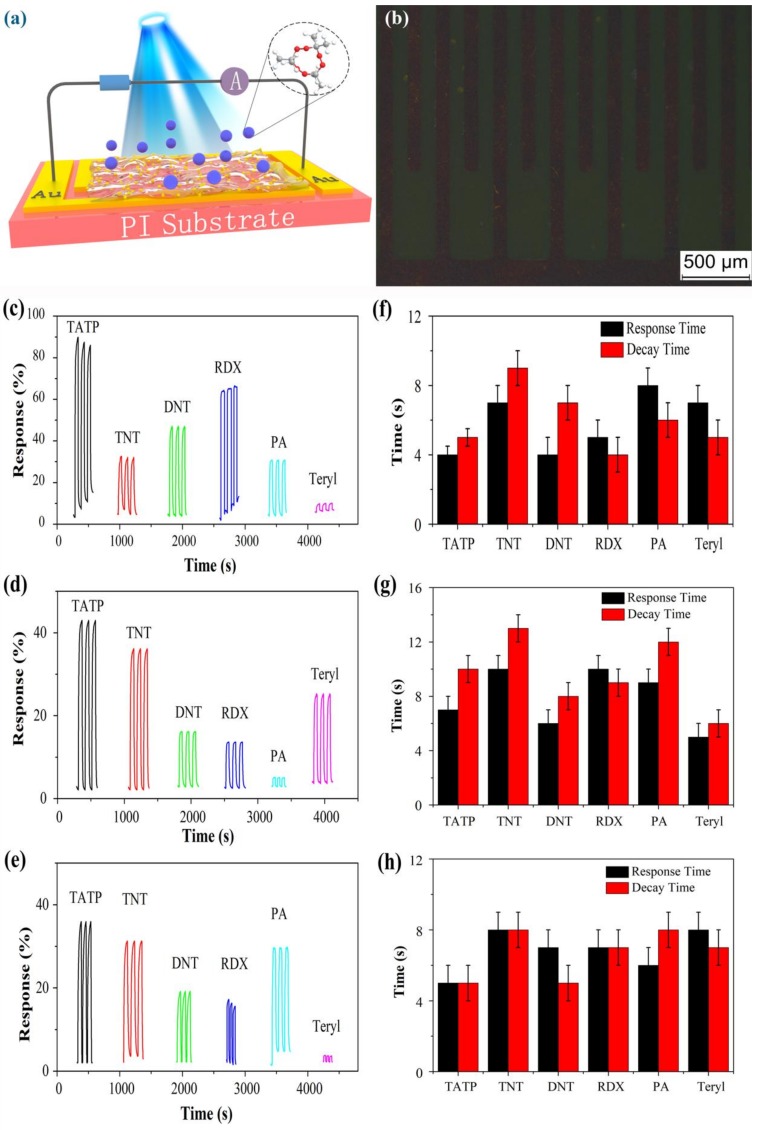
(**a**) Schematic diagram of a sensor film constructed from TiO_2_/PW_11_ nanocrystals. (**b**) Microstructure of interdigital gold electrodes on PI substrate. Response of TiO_2_/PW_11_ to different explosives under (**c**) 365 nm, (**d**) 450 nm and (**e**) 550 nm illumination. Response time and decay time of TiO_2_/PW_11_ sensor film under (**f**) 365 nm, (**g**) 450 nm and (**h**) 550 nm illumination.

The sensing properties of the TiO_2_/PW_11_ sensor film (Figure 6a) to different explosives vapor (TATP, TNT, DNT, RDX, PA and tetryl, respectively) were evaluated under different illumination wavelengths (Figure 6c: 365 nm, Figure 6d: 450 nm, and Figure 6e: 550 nm). The TiO_2_/PW_11_ sensor film was coated onto the interdigital gold electrodes. The electrodes were obtained by electrochemical processing on a flexible PI substrate, and, as shown in Figure 6b, the line width and spacing were 95 μm and 115 μm. Response values of TiO_2_/PW_11_ sensor film to different explosives vapor under different illumination wavelengths are summarized in Table 1. One can see from Figure 6 that, for example, the response (defined as (I_t_ − I_0_)/I_0_) of TiO_2_/PW_11_ sensor film to TATP under 365, 450 and 550 nm illumination was 84%, 42%, and 37%, respectively. It is obvious that the responses of TiO_2_/PW_11_ sensor film toward TATP were larger than those of other explosives under different illumination wavelengths. What is in strong contrast is that, as shown in Figure S1, the responses of pure TiO_2_ toward various explosives were all below 1% under 365 nm illumination. No obvious response could be detected for pure TiO_2_ toward all tested explosives under the illuminations of 450 nm and 550 nm. This result reveals that POMs played a vital role to deliver photogenerated electrons in TiO_2_.The excellent response of TiO_2_/PW_11_ sensor film to TATP may be roughly attributed to the absorption of TATP on the surface of TiO_2_/PW_11_, coupled with photocatalytic decomposition of TATP to acetone and H_2_O_2_. Under the irradiation of light, the photoelectron in conduction band (CB) of TiO_2_ transfers to HOMO of POMs. Thus, the photocurrent from the TiO_2_/PW_11_ system promoted the photocatalytic activity of sensor film. Consequently, the sensor film had better performance when it was under the conditions of ultraviolet and visible light. 

**Figure 7:**
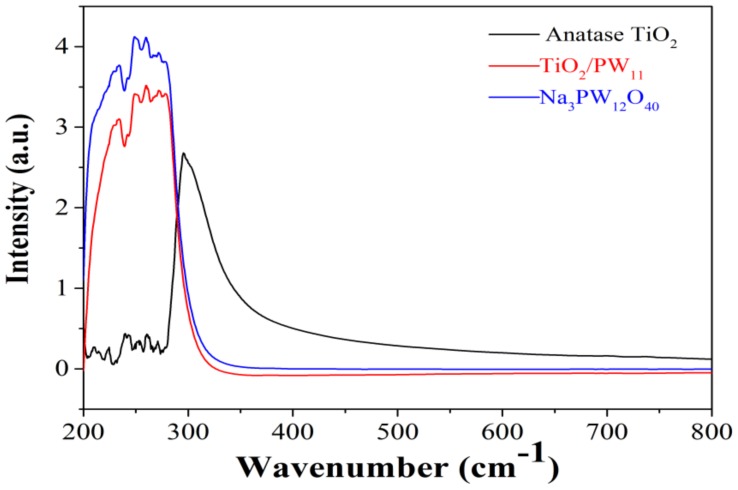
UV-visible spectrum of Na_3_PW_12_O_40_ (blue line), anatase TiO_2_ (black line) and TiO_2_/PW_11_ (red line).

Additionally, as depicted in Figure 6f–h, the response times of TATP were 4, 7, and 5 s while the decay times of TATP were 5, 10, and 5 s, respectively. The response time and decay time of other explosives were all within 10 s. This fast reaction process originates from the ability of POMs to deliver electrons. To demonstrate the role of POMs doped in TiO_2_, the UV-visible spectra of Na_3_PW_12_O_40_, anatase TiO_2_ and TiO_2_/PW_11_ was presented in Figure 7. Compared with the UV absorption at 305 nm of anatase TiO_2_, the absorption of TiO_2_/PW_11_ at 305 nm was dismissed, which may originate from the strong ability of POMs to bear electrons and deliver photoelectrons of TiO_2_, and thus the photoinduced charge carrier’s separation efficiency was improved [32,33]. 

**Figure 8:**
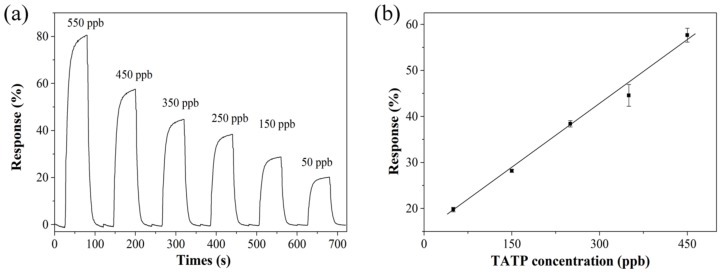
(**a**) The response measured for the TiO_2_/PW_11_ exposed to decreasing concentrations of TATP under 365 nm illumination. (**b**) The calibration curve based on the data from (**a**), the slope of the plot is 0.09 and R-square = 0.99.

Figure 8a presents the response of the TiO_2_/PW_11_ when introduced to vapors of TATP at variable concentrations under 365 nm illumination. TATP was diluted using nitrogen from 550 ppb to 50 ppb. It was difficult to obtain concentrations below 50 ppm with high enough accuracy under our current experimental conditions and therefore the lowest concentration measured was 50 ppb of TATP. The TiO_2_/PW_11_ showed an obvious response of about 20% when the TATP concentration was lowered to 50 ppm, which is lower than that of hybrid organic-semiconductor sensors [26]. Figure 8b shows the liner response of TiO_2_/PW_11_ to TATP vapor at concentrations from 450 to 50 ppm. In addition, the response of the sensor decreased in the range of 57.67%–19.75%.

**Figure 9:**
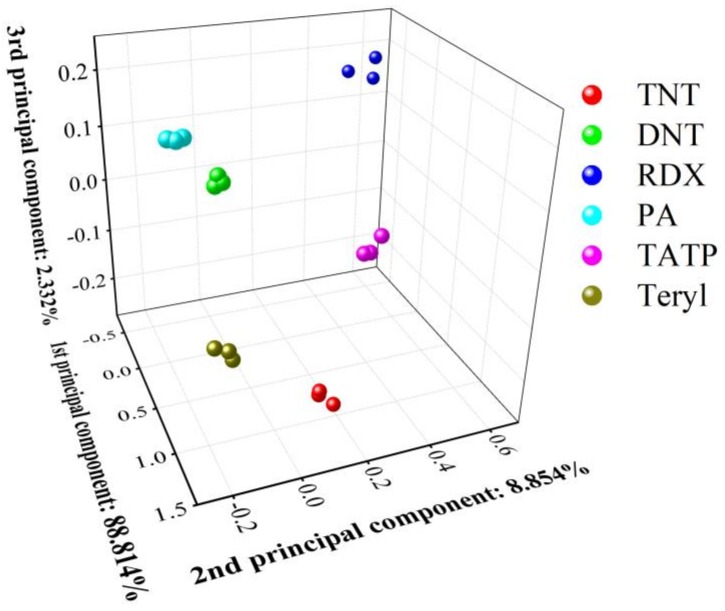
The principal component analysis (PCA) plot of the response values.

Generally, optoelectronic sensing shows great advantages for the high sensitivity in the field of gas sensing. However, a single sensor film can respond to different explosives vapor but cannot discriminate them from each other. The sensor array was constructed using various materials to discriminate target molecules using the statistical procedure in previous work [26]. Here, a sensor array based on the above single optoelectronic TiO_2_/PW_11_ sensor was constructed by regulating the excitation wavelength. Principal component analysis (PCA) was employed to evaluate the recognition capability of the sensor array. This process has been described by Brady, where MATLAB was used to perform the PCA and classification [34]. As shown in Table 1, 54 characteristic response parameters (response, response time and decay time under 365, 450 and 550 nm illumination) were integrated for all the data. A training set of three runs for each explosive (18 runs total; 18 × 54 matrix) was subjected to PCA, yielding a 54 × 54 matrix containing the eigenvectors for the data set. Figure 9 shows the projection of the training data using three of the six principal components for the six explosives. The results obtained from the PCA showed that the cluster of six different explosives congregated separately and independent of each other, allowing for accurate classification. Therefore, the sensor array based on a single TiO_2_/PW_11_ can well distinguish TATP from six different explosives.

**Figure 10:**
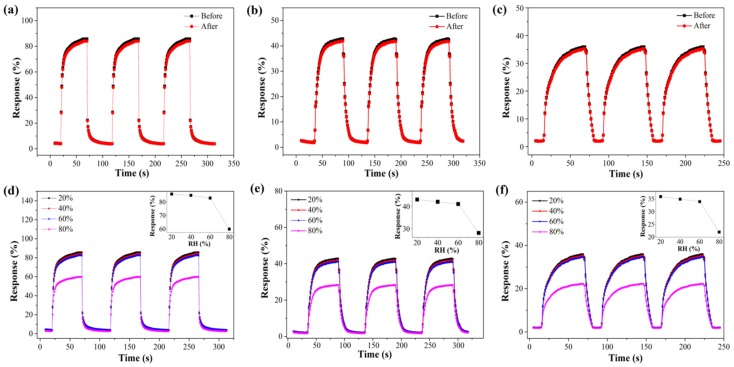
The response of the sensor film to TATP before and after bending under the illumination of 365 nm (**a**), 450 nm (**b**) and 550 nm (**c**). Effect of the humidity under the illumination of 365 nm (**d**), 450 nm (**e**) and 550 nm (**f**).

Furthermore, the flexibility and stability of the flexible sensor film was demonstrated with the bending test. As shown in Figure 10a–c, no significant decrease in the response of sensor film of TATP was found compared to the relaxed state, down to a curvature of 15 mm under the illumination of 365 nm, 450 nm and 550 nm. Repetitive bending (for 50 cycles) did not degrade the sensor performance, which suggests good flexibility and mechanical endurance of the sensor film.

Metal oxide semiconductor nanomaterial is always sensitive to humidity. Many works have developed various methods to minimize the effect of humidity on explosive sensing [35]. Here, the role of relative humidity (RH) is addressed in Figure 10d–f. The response ratios of TATP are almost unchanged, at 20%, 40% and 60% RH under the illumination of 365 nm, 450 nm and 550 nm. However, the response ratio of TATP decreased at 80% RH due to competitive adsorption of TATP and water molecules on the surface of TiO_2_/PW_11_ film. This result illustrates that the sensing response cannot be affected by ambient air below the 60% RH. 

The long-term stability of the flexible sensor film was also studied (Figure S2). The TiO_2_/PW_11_ film has high stability and did not degrade the response to TATP for at least five months under the illumination of 365 nm, 450 nm and 550 nm. Thus, the TiO_2_/PW_11_ sensor film showed excellent long-term stability to TATP, which shows potential for practical usage.

## 4. Conclusions

Keggin structure PW_11_ doped TiO_2_/PW_11_ was constructed by in situ doping of Na_3_PW_12_O_40_ into TiO_¬2_. The function of a sensor array toward an improvised explosive, TATP, can be realized by employing only a TiO_2_/PW_11_ sensor by adjusting the illumination wavelength. The TATP vapor sensing property of the sensor film significantly depended on the illumination wavelength. The remarkable improvement in gas sensing performance under light illumination was attributed to the Keggin POM structures on the surface of TiO_¬2_ because the POMs have a strong ability to bear and deliver electrons. Thus, the number of electrons that participate in the reaction with TATP gas molecules greatly increases. We expect that this study will shine light on the realization of portable, real-time, and cheap platforms for contactless discrimination of explosive monitoring.

## Figures and Tables

**Table 1:** Response value of TiO_2_/PW_11_ sensor film to different explosives vapor under different illumination wavelengths.

Illumination Wavelengths	365 nm			450 nm			550 nm		
Response Value	R ^1^ (%)	R T ^2^ (s)	D T ^3^ (s)	R (%)	R T (s)	D T (s)	R (%)	R T (s)	D T (s)
TATP	81	4	5	42	7	10	37	5	5
TNT	37	7	9	37	10	13	32	8	8
DNT	45	4	7	18	5	8	19	7	5
RDX	69	5	4	14	10	9	17	7	7
PA	32	8	6	4	9	12	30	6	8
Tetryl	8	7	5	28	4	6	2	8	6

^1^ Response (defined as (I_t_ − I_0_)/I_0_). ^2^ Response time. ^3^ Decay time.

## Supplementary Materials

The following are available online at https://www.mdpi.com/1424-8220/19/4/915/s1.
