# Supplementary material for: A Sensor Array Realized by a Single Flexible TiO2/POMs Film to Contactless Detection of Triacetone Triperoxide
Source: Sensors (Basel). 2019 Feb 21;19(4):915. doi: 10.3390/s19040915 (PMC6413185; doi:10.3390/s19040915)
Supplement: Supplementary file 1 [file sensors-19-00915-s001.pdf]

sensors

MDPI

Supporting information

A Sensor Array Realized by a Single Flexible TiO_2/POMs Film to Contactless Detection of Triacetone Triperoxide

Xiaorong Lü [1], Puqi Hao [1], Guanshun Xie [1], Li Gao [1,*], Junyuan Duan [2,*] and Bingxin Liu [1,*]

1 Qinghai Provincial Key Laboratory of New Light Alloys, Qinghai Provincial Engineering Research Center of High Performance Light Metal Alloys and Forming, Qinghai University, Xining 810016, China; jwplxr@sina.com (X.L.); pheghost@outlook.com (P.H.); guanshunxie@126.com (G.X.)

2 State Key Laboratory of Material Processing and Die & Mould Technology, School of Material Sciences and Engineering, Huazhong University of Science and Technology (HUST), Wuhan 430074, China

* Correspondence: love_lier@163.com (L.G.); junyuanduan@sina.com (J.D.); liubx408@nenu.edu.cn (B.L.); Tel.: +86-1869-723-5043 (B.L.)

Table S1. The available techniques for detection of TATP.

Detection techniques	Sensing materials	Limits of detection	Response time	Ref
Semiconductor based vapor sensor	organic-semiconductor sensors	<100 ppb	30 s	[1]
	SnO_2 and WO_3	12 ppb	-	[2]
	metal oxide catalyst	8 ppm	2 min	[3]
	In_2O_3 nanoparticles	2.9 ppb	120 s	[4]
Ion mobility spectrometry	-	1.2 ng	-	[5]
Colorimetric method	colorimetric sensor	< 2 ppb	-	[6]
	silver nanoparticles	20 nM	-	[7]
	N,N-dimethyl-p-phenylene diamine	0.1 mg·L^{-1}	-	[8]
Electrochemical method	Molecularly-imprinted polymer	26.9 μg·L^{-1}	-	[9]
	$Fe^{II/III}$ ethylenediaminetetraacetat e	0.89	-	[10]

Table S2. The atomic percentage results of TiO_2/PW_{11} from XPS.

Name	Peak BE	FWHM eV	Area(P) CPS.eV	Atomic %
**W4f**	35.39	1.19	61905.31	3.94
**Ti2p**	458.56	1.15	123995.77	17.6
**O1s**	530.04	1.47	154800.67	52.12
**P2p**	133.1	0.38	2507.5	1.38

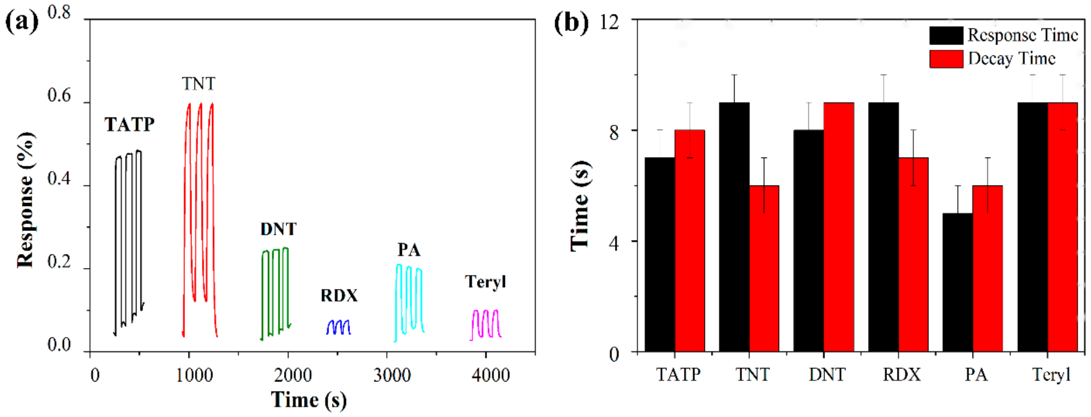

Figure S1. Response and response time and decay time of TiO_2 under 365 nm illumination.

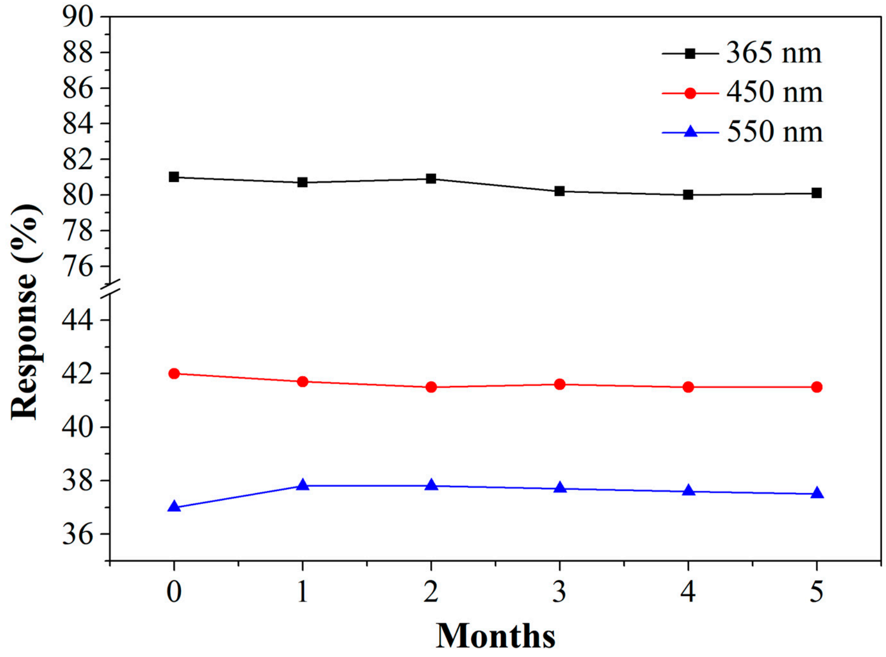

Figure S2. The long-term stability of sensor film under the illumination of 365 nm, 450nm and 550nm.

Refrences

1. Capua, E.; Cao, R.; Sukenik, C.N.; Naaman, R. Detection of triacetone triperoxide (TATP) with an array of sensors based on non-specific interactions. *Sens. Actuators B Chem.* **2009**, *140*, 122–127.
2. Warmer, J.; Wagner, P.; Schöning, M.J.; Kaul, P. Detection of triacetone triperoxide using temperature cycled metal-oxide semiconductor gas sensors. *Phys. Status Solidi* **2015**, *212*, 1289–1298.
3. Amani, M.; Chu, Y.; Waterman, K.L.; Hurley, C.M.; Platek, M.J.; Gregory, O.J. Detection of triacetone triperoxide (TATP) using a thermodynamic based gas sensor. *Sens. Actuators B Chem.* **2012**, *162*, 7–13.
4. Chen, W.-H.Z. and W.-D.Z. and L.-Y. Highly sensitive detection of explosive triacetone triperoxide by an In 2 O 3 sensor. *Nanotechnology* **2010**, *21*, 315502.
5. Wen, M.; Jiang, L.; Liu, W.; Cheng, S.; Wang, W.; Chen, C.; Liang, X.; Zhou, Q.; Peng, L.; Li, J.; et al. Sensitive Detection of Triacetone Triperoxide (TATP) by Acetone-Assisted Photoionization Ion Mobility Spectrometry. *J. Chinese Mass Spectrom. Soc.* **2014**, *356*, 481–487.

6. Lin, H.; Suslick, K.S. A Colorimetric Sensor Array for Detection of Triacetone Triperoxide Vapor. **2010**, *132*, 15519–15521.
7. Üzer, A.; Durmazel, S.; Erçağ, E.; Apak, R. Determination of hydrogen peroxide and triacetone triperoxide (TATP) with a silver nanoparticles—based turn-on colorimetric sensor. *Sens. Actuators B Chem.* **2017**, *247*, 98–107.
8. Can, Z.; Üzer, A.; Türkekul, K.; Erçağ, E.; Apak, R. Determination of Triacetone Triperoxide with a N,N-Dimethyl-p-phenylenediamine Sensor on Nafion Using Fe_3O_4 Magnetic Nanoparticles. *Anal. Chem.* **2015**, *87*, 9589–9594.
9. Mamo, S.K.; Gonzalez-rodriguez, J. Development of a Molecularly Imprinted Polymer-Based Sensor for the Electrochemical Determination of Triacetone Triperoxide (TATP). *Sensors* **2014**, *14*, 23269–23282.
10. Laine, D.F.; Roske, C.W.; Cheng, I.F. Electrochemical detection of triacetone triperoxide employing the electrocatalytic reaction of iron(II/III)-ethylenediaminetetraacetate and hydrogen peroxide. *Anal. Chim. Acta* **2008**, *608*, 56–60.
